# A Comparative Review of Equine SIRS, Sepsis, and Neutrophils

**DOI:** 10.3389/fvets.2019.00069

**Published:** 2019-03-12

**Authors:** M. Katie Sheats

**Affiliations:** Department of Clinical Sciences, North Carolina State University College of Veterinary Medicine, Raleigh, NC, United States

**Keywords:** sepsis, systemic inflammatory response, neutrophil, endotoxemia, organ dysfunction

## Abstract

The most recent definition of sepsis in human medicine can be summarized as organ dysfunction caused by a dysregulated host response to infection. In equine medicine, although no consensus definition is available, sepsis is commonly described as a dysregulated host systemic inflammatory response to infection. Defense against host infection is the primary role of innate immune cells known as neutrophils. Neutrophils also contribute to host injury during sepsis, making them important potential targets for sepsis prevention, diagnosis, and treatment. This review will present both historical and updated perspectives on the systemic inflammatory response (SIRS) and sepsis; it will also discuss the impact of sepsis on neutrophils, and the impact of neutrophils during sepsis. Future identification of clinically relevant sepsis diagnosis and therapy depends on a more thorough understanding of disease pathogenesis across species. To gain this understanding, there is a critical need for research that utilizes a clearly defined, and consistently applied, classification system for patients diagnosed with, and at risk of developing, sepsis.

## Introduction—the Impact of Sepsis

The Society of Critical Care Medicine and the European Society of Critical Care Medicine task force define sepsis as life-threatening organ dysfunction caused by a dysregulated host response to infection (1). Sepsis claims millions of human lives worldwide each year, and in North America alone, more than 750,000 people develop sepsis annually (2). While the sepsis mortality rate in humans appears to be declining (27% in 2007, down from 39% in 2000) (3), the continued rise in the rate of sepsis diagnosis, which reportedly doubled between 2000 and 2008 (4), made it the 11th leading cause of death in the United States in 2010 (5). From a national health perspective, the impact of sepsis is significant, as the Healthcare Cost and Utilization Project (HCUP) identified sepsis as the single most expensive condition treated in hospitals in 2011, with a total aggregated cost of $24 billion (6). Because of its impact, sepsis continues to be the focus of intense research in which the current understanding of pathophysiology is being reevaluated, the relevance of various *in vitro* and *in vivo* models is being challenged (7–9) and the importance of species-specific differences is being recognized (10). With advanced information and understanding, clinicians, and basic scientists will be able to develop new approaches and new targets for the treatment and even prevention of this devastating condition.

While there are still fewer reports on the cost and incidence of sepsis in horses compared to humans, significant progress has been made in recent years to better understand the impact of sepsis diagnosis on equine patient outcomes, particularly in foals. Sepsis is one of the most common reasons for neonatal foals to present to tertiary care veterinary hospitals (11, 12). In a recent retrospective study, Giguere et al. reported on the primary and secondary diagnoses of 1,065 equine neonates ≤ 14 days of age presented to an intensive care unit (ICU) between 1982 and 2008 (13). These authors report that 453 of the 1,065 foals (42.5%) had a positive blood culture, and 641 of the 1,065 foals (60.2%) were classified as septic. In this study, sepsis was defined as any or all of the following criteria: (1) positive blood culture, (2) more than 1 site of infection *ante mortem*, or (3) *post mortem* evidence of more than 1 septic process. One of the more interesting findings to come from this research is the evidence that survival of foals admitted to neonatal ICUs, although not specifically for sepsis, has increased significantly over the past 3 decades. In another multicenter study of hospitalized equine neonates, Wong et al. reported that 147 of 273 (46%) foals ≤ 30 days of age were classified as septic (14). Foals in this study were classified as septic based on the same criteria reported by Weber et al. (15). Wong et al. reported that 73% (92 of 126) of septic foals in their study survived to discharge (14). Overall, reported survival rates for foals with sepsis varies from 45–81%, with significant variability in sample population and sepsis definition between studies (16–22). In terms of financial cost, one prospective study reported that the mean cost of hospitalization and treatment for foals that survived sepsis was $2842.00 (23); but based on severity of illness and duration of hospitalization, the individual patient costs can be much higher.

In contrast to the larger number of studies that have examined the impact of sepsis on survival in hospitalized foals, studies on sepsis mortality in adult horses are rare. In 2017, Arroyo et al. reported on factors associated with survival in 97 horses with septic pleuropneumonia (24). In this paper, sepsis was defined as the presence of systemic inflammatory response syndrome (SIRS) and a positive bacterial culture from a tracheal aspirate or pleural fluid. Sixty-five of the 97 horses (67%) with septic pleuropneumonia survived to discharge. Other recent studies on mortality of hospitalized adult horses have chosen to examine outcomes in patients with diagnoses other than sepsis, including endotoxemia (25, 26), SIRS (27, 28) and multiple organ dysfunction syndrome (MODS) (29, 30). Until consensus definitions are available to equine practitioners, the impact of sepsis on survival in adult horses is likely to remain unknown (31).

## Defining Sepsis

In 1991, Roger C. Bone chaired a “Consensus Conference” of the American College of Chest Physicians (ACCP) and the Society of Critical Care Medicine (SCCM), which was tasked with the “goal of agreeing on a set of definitions that could be applied to patients with sepsis and its sequelae” (32). It was expected that the definitions developed would be broad enough to improve early bedside detection of sepsis in order to allow for early therapeutic intervention. It was also anticipated that consensus on sepsis definitions would help to standardize research protocols, which would lead to “improved dissemination and application of information derived from clinical studies” (33). The result was the development of a broad series of definitions (Table 1) that is still used in practice today and has served as the basis for numerous human clinical trial inclusion criteria over the past two decades (34).

**Table 1:** ACCP/SCCM consensus conference definitions (1991).

**ACCP/SCCM consensus conference definitions**
**Sepsis—**The clinical syndrome defined by the presence of both infection and a systemic inflammatory response.
**Severe sepsis—**Sepsis associated with organ dysfunction, hypoperfusion, or hypotension. Hypoperfusion and perfusion abnormalities may include, but are not limited to lactic acidosis, oliguria, or an acute alteration in mental status.
**Septic shock—**Sepsis-induced with hypotension despite adequate fluid resuscitation along with the presence of perfusion abnormalities that may include, but are not limited to lactic acidosis, oliguria, or an acute alteration in mental status. Patients who are receiving inotropic or vasopressor agents may not be hypotensive at the time that perfusion abnormalities are measured.
**Multiple organ dysfunction syndrome—**Presence of altered organ function in an acutely ill patient such that homeostasis cannot be maintained without intervention.

*Adapted from Bone et al. (32)*.

Importantly, as a result of this conference the term “systemic inflammatory response syndrome” (SIRS) was put forward as a criterion of the sepsis definition. SIRS was described as a non-specific, clinical, pro-inflammatory immune response. While it was understood at the time that SIRS can occur in response to numerous events that injure host tissue (i.e., trauma, anaphylaxis, ischemia, hemorrhage), in the case of sepsis, the inciting event for SIRS is documented or suspected infection. As defined by Bone et al. SIRS is present when human patients experience 2 or more of the following *acutely altered* physiologic changes, in the absence of other known cause for such abnormalities:

- Body temperature > 38°C (100.4°F) or < 36°C (96.8°F)
- Heart rate > 90 beats/min
- Hyperventilation (respiratory rate > 20 breaths/min or PaCO2 < 32 mmHg)
- White blood cell count > 12,000 cells/μl or < 4,000 cells/μl or > 10% band forms

The definition of SIRS proposed by Bone et al. along with the definitions of “sepsis,” “severe sepsis” and “septic shock,” has also gained general acceptance within veterinary medicine and are cited in equine specific-reviews on sepsis (35, 36). Although no consensus definition of sepsis in veterinary species is currently available, examples of published criteria used to diagnose SIRS include two or more of the following in adult horses (37):

- Body temperature > 38.6°C (101.5°F)
- Heart rate > 60 beats/min
- Hyperventilation (respiratory rate > 30 breaths/min)
- White blood cell count > 12,500 cells/μl or < 4,500 cells/μl and 10% band neutrophils

and two or more of the following in equine neonates (18):

- Body temperature > 39.2°C (102.6°F) or < 37.2°C (99°F)
- Heart rate > 120 beats/min
- Hyperventilation (respiratory rate > 30 breaths/min)
- White blood cell count > 12,900 cells/μl or < 4,000 cells/μl, or > 10% band neutrophils
- and evidence of sepsis, cerebral ischemia or hypoxia, or trauma

Using these criteria, researchers determined that SIRS was present in 30% of adult horses with colic (37) and more than 40% of critically ill neonatal foals (18) who presented to a teaching hospital for evaluation and treatment.

In 2001, a second International Sepsis Definitions Conference convened to respond to growing criticisms from experts in the field that the 1991 ACCP/SCCM definitions did not reflect an up-to-date understanding of the pathophysiology of human sepsis and its associated syndromes and were contributing to erroneous and flawed clinical trial data (38–42). Critics were primarily dissatisfied with the inclusion of the SIRS criteria in the definition of sepsis, arguing that the criteria lacked specificity and were too sensitive to be of use in clinical diagnosis or clinical trials (41). This argument was supported by data showing that most ICU patients and many general ward patients met the SIRS criteria (43–46). Further criticism claimed that a SIRS diagnosis provided no information regarding the underlying disease process and was simply a list of clinical signs routinely seen in patients having an appropriate response to a physiologic insult. Another important criticism of the sepsis definition was that actual infection could often only be identified in 50% (or fewer) of patients who “appeared” septic (7, 44, 47).

Given the list of complaints leveled against the 1991 ACCP/SCCM sepsis definitions, it is somewhat surprising that very few changes to the original definitions were made by the second Sepsis Definitions Conference (Table 2). The participants of the 2001 conference did concur that the SIRS criteria were too sensitive and lacked needed specificity. As a result, they created an expanded list of possible signs of systemic inflammation in response to infection (Table 3), with the caution that practitioners looking to establish a diagnosis of sepsis should only include those diagnostic criteria that cannot be easily explained by other causes. The 2001 Consensus concluded that more meaningful changes to the definition of sepsis and its associated conditions based on biomarkers were premature and that future changes to these definitions would rely on an increased understanding of—and readily available means to diagnose—the immunological and biochemical processes involved in sepsis (34).

**Table 2:** SCCM/ESISM/ACCP/ATS/SIS sepsis definitions (2001).

**SCCM/ESICM/ACCP/ATS/SIS sepsis definitions**
**Sepsis—**The presence of infection, documented or strongly suspected, with a systemic inflammatory response.
**Severe sepsis—**Sepsis complicated by organ dysfunction.
**Septic shock—**Severe sepsis complicated by acute circulatory failure characterized by persistent arterial hypotension, despite adequate volume resuscitation, and unexplained by other causes.
**Multiple organ dysfunction syndrome—**Presence of altered organ function in an acutely ill patient such that homeostasis cannot be maintained without intervention.

*Adapted from Levy et al. (34)*.

**Table 3:** SCCM/ESISM/ACCP/ATS/SIS diagnostic criteria for sepsis (2001).

**SCCM/ESICM/ACCP/ATS/SIS Expanded diagnostic criteria for sepsis**
**Documented or suspected infection and some of the following:**
**General parameters:** Fever Hypothermia Tachycardia Tachypnea Altered mental status Significant edema or positive fluid balance Hyperglycemia (in the absence of diabetes)
**Inflammatory parameters:** Leukocytosis Leukopenia Normal white blood cell count with > 10% immature forms Plasma C reactive protein >2 SD above normal value Plasma procalcitonin >2 SD above the normal value
**Hemodynamic parameters:** Arterial hypotension Mixed venous oxygen saturation >70% Cardiac index >3.5 1 min^−1^ m^−2^
**Organ dysfunction parameters:** Arterial hypoxemia Acute oliguria Creatinine increase Coagulation abnormalities Ileus Thrombocytopenia Hyperbilirubinemia
**Tissue perfusion parameters:** Hyperlactatemia Decreased capillary refill or mottling

*Adapted from Levy et al. (34)*.

## Staging Sepsis, Modeling Equine Patient Outcomes

The concept of a clinically useful sepsis staging system was introduced by the 2001 International Sepsis Definitions Conference. The consensus stated that such a system would “stratify patients by both their risk of an adverse outcome and their potential to respond to therapy” (48). The classification scheme proposed for sepsis—called PIRO [Predisposition, Insult, Response, Organ failure (PIRO)]—addressed 4 major areas to stage sepsis in a manner similar to the TNM (Tumor, Lymph Nodes, Metastasis) staging system ((237)) used for oncology patients. The first component, **Predisposition**, addresses the growing understanding that the same insult (i.e., infection) can cause a more severe response or worse outcome in some individuals compared to others. In human medicine, the variables of “predisposition” include genetics, gender, age, and nutritional status. Clinically, this category is also recognized in veterinary patients. Nemzek et al. demonstrated that the white blood cells of Rottweilers and Doberman Pinschers produce a marked inflammatory cytokine response compared with that of mixed breed dogs (49). The authors correlate their findings with the predisposition of these breeds for severe infection with canine parvovirus. The concept of Predisposition is also relevant to equine patients, whose white blood cells (compared with other species) demonstrate a markedly pro-inflammatory response to lipopolysaccharide (LPS) (35, 50). Additionally, an equine experimental model shows that gene expression in LPS-stimulated peripheral blood mononuclear cells is distinctly different in some horse “families” compared to others (51). Further “predispositions” that would be interesting to investigate in terms of equine sepsis could include diseases that predispose horses to dysregulation of insulin, glucose or cortisol, such as Equine Metabolic Syndrome (EMS) or Pituitary Pars Intermedia Dysfunction (PPID). Investigators have already shown that free cortisol fraction is significantly increased in healthy overweight horses, as well as horses with PPID and insulin dysregulation (52). In a prospective observational study, Hinchcliff et al. reported that higher serum cortisol concentrations were significantly associated with increased risk of non-survival in a group of 35 adult horses presented for colic (53). While SIRS status was not reported in the Hinchcliff study, Roy et al. recently reported that SIRS at presentation was associated with increased odds of death in a group of 247 adult horses presented for colic (27), suggesting that non-surviving horses with high cortisol in the Hinchcliff study also had SIRS. Other investigators determined that non-surviving adult hospitalized horses with SIRS had significantly decreased glucocorticoid binding affinity (54). Dysregulation of cortisol has already been associated with poorer sepsis outcomes in human patients. In a study on 164 pediatric patients, Alder et al. reported that patients with both low glucocorticoid receptor expression and high serum cortisol had higher rates (75%) of multi-organ (>2) failure and death (55). Taken together, this evidence suggests that previous EMS and/or PPID diagnosis could be a relevant “predisposition” for horses being treated for sepsis, and outcomes in this patient population may warrant further investigation.

The second component of the PIRO staging system addresses **Insult** (i.e., infection). This category conveys the importance of infection type and location, which is known to have an impact on outcome in both humans and horses (56–58). A recent publication by Declue et al. demonstrates that equine blood stimulated with components of Gram-negative and Gram-positive organisms produces differing cytokine profiles (59). Other recent studies on equine sepsis in both foals and adults examine the impact of Gram-positive and mixed infections on clinicopathologic features of disease and outcomes (16, 60–63). Beyond infection, “insult” could also stratify patients based on primary disease. While reported causes of “insult” leading to sepsis in adult horses include only a few causes such as pneumonia and colitis (24, 63), “insult” in equine neonates is highly varied and includes primary diagnoses such as ruptured bladder, pre-/dysmaturity, failure of passive transfer, neonatal isoerythrolysis, neonatal maladjustment syndrome, etc. While many studies describe blood culture as the gold standard for providing evidence of infection-related SIRS (64), other recent studies have chosen to also accept clinical evidence of infection plus SIRS as supportive of sepsis diagnosis (15, 18). Given the relatively low sensitivity of blood culture as used in veterinary medicine, it seems likely that blood culture as a stand-alone gold standard would lead to under-diagnosis of sepsis and that an “either/or” definition will have the most utility in the context of equine medicine (31).

The third component of the PIRO sepsis staging system is the **Response** to sepsis. The physical and biochemical parameters that routinely describe septic human and veterinary patients are well-known and incorporated into the currently accepted definitions of sepsis and its associated syndromes (32, 34). Unfortunately, the clinical application of these parameters (i.e., SIRS diagnosis) often fails to help clinicians gauge the severity of, or the prognosis for, an individual patient's illness; additionally, they lack sensitivity when it comes to inclusion criteria for clinical trials. Biomarkers of sepsis, in conjunction with other criteria, are increasing in their utility for diagnosing, guiding treatment and predicting outcomes for patients with sepsis (65–69). Specific biomarkers that have recently shown promise in prognosticating human sepsis include serum procalcitonin (PCT), N-terminal brain natriuretic propeptide (NT-proBNP), interleukin-6 (IL-6), prothrombin time (PT), and thrombin time (TT) (70, 71). Procalcitonin is also used to assist in selection of empirical antimicrobial therapy, as this biomarker has been shown to be significantly higher in patients with Gram-positive sepsis compared to Gram-negative sepsis (72, 73). Circulating biomarkers of endothelial cell dysfunction, including circulating adhesion Angiopoietin-2/Angiopoietin-1 ratio (Ang-2/Ang-1) and Angiopoietin-1/Tie-2 ratio (Ang-1/Tie-2), intercellular adhesion molecule (ICAM)-1, vascular cell adhesion molecule (VCAM)-1 and thrombomodulin (TM), have also shown to be independent predictors of 90-day mortality in ICU patients with severe sepsis and septic shock (74). C-reactive protein is also one of the most widely studied human sepsis biomarkers, although evidence suggests that it is more useful for predicting fatal progression of septic patients rather than aiding in initial sepsis diagnosis (75). Biomarkers of inflammation and sepsis investigated in foals and adult horses include lactate, soluble CD14 (sCD14), serum amyloid A, procalcitonin, C-reactive protein (CRP), haptoglobin (Hp), interleukin 1β (IL-1β), interleukin-10 (IL-10), and interleukin-6 (IL-6) (25, 48, 76–80). In a prospective study of 643 foals, median L-lactate concentration in septic foals (4.8 mmol/L) was significantly higher than that of non-septic foals (3.3 mmol/L) (48). In 95 adult horses presented for emergency, Roy et al. report that plasma blood lactate >2.06 mmol/L significantly increased the likelihood of death (OR = 5.65; 95% CI, 2.38–14.13, *P* < 0.001), and lactate was included in their final outcome model of severe SIRS (27). In multiple studies, sCD14 has been shown to be elevated in either foals with septicemia (81) or adult horses classified as sick (82), or clinically endotoxemic (25); however, sCD14 has been demonstrated as a poor predictor of outcome (82). Procalcitonin, which is one of the most widely used sepsis biomarkers in human medicine, has only recently been investigated in horses. Using an ELISA they developed, Rieger et al. reported that mean plasma procalcitonin levels were significantly different between septic (*n* = 5) and healthy (*n* = 24) horses (8,450 ng/mL vs. 47 ng/mL) (76). In a multicenter, prospective, observational study of < 24 h old septic (*n* = 40), sick non-septic (*n* = 40) and < 7 day old healthy foals, Zabrecky et al. reported that while plasma CRP increases with inflammation in neonatal foals, neither CRP or Hp appear to be useful as single time point biomarkers for sepsis in foals (77). Burton et al. reported that the serum IL-6:IL-10 ratio could prove valuable as a prognosticator for equine neonatal septicemia (80). Using white blood cell gene expression analysis, Castagnetti et al. determined that IL-1β is significantly elevated in < 7 day old sick foals with sepsis compared with non-septic sick foals and healthy foals (79). In a carbohydrate overload model of equine sepsis designed to induce laminitis, Steelman et al. used network and correlation analysis of serum metabolomics and determined that the amino acid citrulline was significantly lower in all septic study animals. As part of the same study, Steelman et al. showed that citrulline levels were significantly lower in clinical patients with adverse outcomes (i.e., laminitis or death) following presentation for sepsis or colic (6 of 19 horses) (83). Citrulline has also been identified as a potential prognostic biomarker for foals with sepsis (84). With improved sepsis definitions and agreement on relevant criterion for clinical investigations, biomarkers will undoubtedly improve the understanding, diagnosis, treatment, and even prevention, of equine sepsis.

The final component of the PIRO staging system is **Organ** failure. Evidence from both human and veterinary medicine indicates that evidence of organ dysfunction is an important determinant of prognosis in patients with sepsis (29, 85–87). Recently, McConachie et al. developed and validated a scoring system for multiple organ dysfunction in adult horses with acute surgical colic (29). Specific criteria were measured to monitor function of the heart (serum cardiac troponin, stroke volume index, standard deviation of normal-to-normal intervals), kidneys (creatinine, Δ creatinine), liver (serum bile acids concentrations), gastrointestinal tract (nasogastric reflux volume, abdominal distension), musculoskeletal system (serum creatine kinase activity, Obel grade lameness), respiratory system (PaO2/FiO2 ratio, respiratory rate, and effort), and coagulation pathway (platelet count or prothrombin time). The neurologic system was evaluated with a modified pain score that included postural and social behaviors, demeanor, and activity. Based on laboratory reference ranges, literature review and clinical judgement, these investigators assigned each organ a score where 0 is unaffected, 1–2 is affected and 3 is failed, yielding a maximum organ failure score of 24. Data for MODS scoring was collected on Day 1 and 2 postoperatively in 62 adult horses presented for colic and exploratory laparotomy. The MODS score generated by these criteria had a very high association with 6-month survival, and an optimal cut point of ≥ 8 resulted in a sensitivity of 92% and a specificity of 88% for predicting survival to 6 months. Additionally, these investigators also determined that the number of organs affected (≥ 1) and the number of organs failed (= 3) were also significantly associated with 6-month survival, and that horses with a score ≥ 8 were 10.7 times more likely to have SIRS. Clearly, this investigation demonstrates the potential clinical utility of a MODS score for hospitalized adult horses. One potential drawback for clinical use of the proposed MODS score would be the added cost to the client for score data that is not already routinely collected, but if early identification and aggressive treatment of at-risk patients could improve patient outcomes, the benefits could ultimately outweigh the costs.

Since its inception, the PIRO concept (Table 4) has proven to be a reliable predictor of mortality in human patients with sepsis (88, 89). It remains to be seen whether the PIRO concept has applications in veterinary medicine.

**Table 4:** P I R O (sepsis patient staging system).

	**P** **Predisposition**	**I** **Infection**	**R** **Response**	**O** **Organ dysfunction**
**Available**	Age Comorbidities Chronic Conditions Baseline severity Source of admission	Pathogen Susceptibility Bacteremia Bacterial load Site of infection Nosocominal or community-acquired	Clinical resolution Hypoxemia Hypotension Immune response	ARDS Shock Acute renal failure MODS SOFA
**Future**	Genetics Polymorphisms of TLRs, IL-1, TNF and CD14	Genotyping Assay of microbial products Detection of virulence factors	Biomarkers	Mitochondrial dysfunction Endothelial damage and activation

*Adapted from Levy et al. (34)*.

## Definitions of SIRS in Equine Medicine

While numerous equine studies have utilized the consensus definitions of SIRS and sepsis proposed by human medicine, there is ongoing debate on how to best define these terms for equine veterinary medicine. Consequently, veterinary researchers have directed significant efforts toward developing and interrogating various SIRS/sepsis scores and models to aid clinicians in predicting patient outcomes.

### SIRS Criteria in Neonates/Foal

In a recent review of equine neonatal sepsis, Wong et al. (90) present criteria for SIRS diagnosis based on the presence of 3 of 6 criteria (fever or hypothermia, tachycardia, tachypnea, leukocytosis, or leukopenia, hyperlactatemia, hypoglycemia) tailored to 4 different age categories (newborn, neonate, juvenile, weanling) (90). Wong et al. further stipulate that, as in human pediatrics (91), at least 1 of the criteria must be abnormal temperature or leukocyte count due to the fact that diseases affecting neonates often cause tachycardia and tachypnea. In a subsequent paper, Wong et al. use their neonatal SIRS criteria to create what they called an “updated” sepsis score (14), which they based on the “modified equine neonatal sepsis score” published in 1988 (92). In addition to adding the neonatal SIRS criteria, the updated sepsis score also incorporated blood L-lactate, serum creatinine and lymphocyte count. Using a prospective study design, the investigators collected historical, physical examination, and clinicopathologic findings on 273 sick foals (<30 days old) presented to six different veterinary referral centers to compare the association of original and updated equine neonatal sepsis and SIRS criteria with sepsis in foals. Foals were classified as septic if they had any one of the following: (1) positive blood culture, (2) >1 site of infection based on cytology, bacterial culture, or histopathology, or (3) postmortem evidence of >1 septic process, as previously described (15). The “updated” sepsis score did not improve the ability to predict sepsis over the modified sepsis score, and the modified SIRS criteria was slightly better at predicting sepsis (more significant OR) than the equine neonatal SIRS criteria (14). In another recent report, Weber et al. conducted a retrospective cohort study on hospital records of equine neonates admitted to a veterinary teaching hospital between 1982 and 2008. Data collected from these records were used to evaluate performance of the “modified sepsis score” (93), to determine whether sepsis scores change significantly over time and to determine objective clinical factors associated with sepsis. Using data from 1065 foals, Weber et al. showed that the modified sepsis score was significantly better at predicting sepsis than a multivariable model generated from objective criteria. They also show that diagnostic performance of the modified sepsis score did not change significantly over time, and that the sensitivity of the modified sepsis score in this patient population was improved by decreasing the cut point from >11 to >7 (15).

### SIRS Criteria in Adult Horses

While several studies have examined SIRS and sepsis criteria in equine neonates, there is only one recent study on SIRS in adult horses. In 2017, Roy et al. conducted a prospective observational study to determine the prognostic value of measures of SIRS in adult horses presented for emergency treatment and to identify the best model of severe SIRS to predict outcome. In this study, SIRS criteria included the following: heart rate >52 bpm, respiratory rate >20 bpm, temperature outside the range of 37.0–38.5°C and WBC count outside the ranges of 5.0–12.5 × 10^9^/L. Using these criteria, Roy et al. reported that 31% of 464 emergency cases had 2 or more abnormal SIRS criteria at admission and that SIRS diagnosis was associated with increased odds of death. They also reported that horses with 3 and 4 signs of SIRS (SIRS3/4) had increased odds of death compared to non-SIRS cases (OR = 19.80; 95% CI, 9.18–42.74, *P* < 0.001) or the horses with just 2 signs of SIRS (SIRS2) (OR = 4.45; 95% CI, 1.78–11.15; *P* = 0.002). Additionally, they determined that a model of severe SIRS, including SIRS score and markers of tissue perfusion (blood lactate concentration >2.06 mmol/L and altered mucus membrane color), generated the best model for predicting outcome in this population of horses (27).

## Mechanisms of Host Defense—Pathophysiology of Sepsis

### Local Inflammation

The four cardinal signs of inflammation (as described in the 1st century AD) are *rubor et tumor cum calore et dolore* (94). This translates to redness and swelling with heat and pain. In today's terms, we explain these ancient observations with the modern terms of peripheral vasodilation (redness), vascular leakage (swelling), and fever (heat). We now understand that these concepts, recognized by the Ancient Greeks Galen and Celsus, are essential to local host defense from invading microorganisms. Within the environment, plants, and animals are exposed to potentially invasive microorganisms, and as such are armed with defensive mechanisms. In mammals, the first line of defense is the physical barrier, including skin and mucous membranes, mucous layers that line the respiratory and gastrointestinal tracts, various enzymes, and antimicrobial peptides. These barriers repel the vast majority of microorganisms, but when pathogens do “breach the wall,” vasodilation and vascular leakage, mediated by the local release of tumor necrosis factor-α (TNFα), interleukin (IL)-1β and histamine, promotes the recruitment of professional phagocytes (i.e., neutrophils) to the area (95). Armed with bactericidal and proteolytic enzymes, as well as the ability to produce reactive oxygen intermediates, neutrophils are uniquely suited for killing pathogens. Additionally, local activation of the coagulation cascade slows the dissemination of the infectious organisms through the formation of microthrombi, which occlude small vessels. Ultimately, the goal of these defense mechanisms is to eliminate the pathogen locally, at the site of invasion. Sepsis occurs when the host response to infection becomes systemic. This systemic response has detrimental effects on tissues and organ systems remote from the site of initial injury (96). The pathophysiology of this process is extremely complex.

### Pathogen Recognition—A Key Component of Host Defense

In vertebrates, there are two arms to the immune system: innate and adaptive immunity. The innate immune system was the first to evolve, and as such is present in all plants and animals, while adaptive immunity is found only in vertebrates. Both components of the vertebrate immune system have unique features that are vital for optimal host defense. The innate immune response is capable of instantaneous pathogen recognition and rapid mobilization of destructive cellular forces, but it is incapable of providing long-term immunity, which is the responsibility of the slower-responding adaptive immune system.

### Triggers and Receptors of the Innate Immune Response

Key molecules involved in the immune system recognition of pathogens and tissue injury are pathogen-associated molecular patterns (PAMPs) and damage-associated molecular patterns (DAMPs, also “alarmins”), respectively. The list of PAMPs includes LPS, flagellin, peptidoglycan, lipoteichoic acid, double stranded viral DNA, and unmethylated CpG motifs (97). Molecules that act as DAMPs include hyaluronan, heparan sulfate, heat shock proteins, high mobility group protein B1 (HMGB1) and adenosine triphosphate (ATP) (98). While DAMPs have received recent attention as models of sterile inflammation, both PAMPs and DAMPs are relevant to the discussion of sepsis pathophysiology. The hallmark of clinical sepsis is a harmful or damaging host response to infection. While it is accepted that PAMPs are involved in the initiation of the inflammatory response, it is reasonable to suspect that tissue injury caused by invading pathogens and/or the host (i.e., neutrophil mediated damage) would also generate DAMPs. DAMPs could then further activate the innate immune response, leading to additional inflammatory cytokine signaling, neutrophil recruitment, and tissue damage. Considering the cross-reactivity of individual pattern recognition receptors (PRRs) for PAMPs and DAMPs (98–100), a vicious cycle of signal amplification ultimately results in a globally dysregulated immune response. Evidence shows that interaction of PAMPs and DAMPs with their receptors is correlated with increased rates of fatality in a polymicrobial mouse model of sepsis (101). Although at present the consequences of this amplification cycle in veterinary species is only inferred, cross-reactivity of equine PRRs with different DAMPs and PAMPs has been demonstrated (59, 102).

The PAMPs and/or DAMPs present in inflamed and/or infected tissues are recognized by signaling PRRs on the surface of immune cells such as neutrophils, macrophages, mast cells, and dendritic cells, and non-immune cells such as epithelial cells, endothelial cells, myocytes, and fibroblasts (100, 103, 104). There are numerous types of PRRs, including leucine-rich repeat-containing proteins (NLRs, previously called NOD-like receptors), C-type lectin receptors (CLRs), RIG-I-like receptors (RLRs), and Toll-like receptors (TLRs) (105). TLR signaling is an essential step in the activation of the innate immune response. TLRs are needed for the clearance of bacterial infection, but excessive TLR signaling can lead to systemic inflammation, which can have detrimental consequences to the host. Because of the relative importance of endotoxin in a number of equine diseases, Toll-like receptor 4 (TLR4), which binds the lipid A component of LPS, has been the subject of much research and will be further discussed. Other members of the TLR family identified in horses (TLR 2, 3, 5, 8, and 9) are reviewed in detail elsewhere (35, 97, 106).

In order to bind TLR4, LPS must first interact with LPS-binding protein to then bind either soluble or membrane-bound (myeloid cells) CD14 (107, 108). After CD14 binding, LPS interacts with TLR4 and its co-receptor MD-2, which results in TLR/interleukin (IL)-1 receptor-associated protein (TIRAP)-dependent recruitment of the MyD88 adaptor protein (109). MyD88 activation causes activation of the transcription factor NF-κB as well as phosphorylation of mitogen-activated protein kinases (MAPK). The most well-recognized and frequently reported result of TLR4-LPS binding is NF-κB-mediated upregulation of pro-inflammatory mediators such as TNFα and IL-1β. However, LPS-mediated TLR4 activation on circulating monocytes in some species induces simultaneous upregulation of IL-10 and IL-1Rα genes, showing that TLR4 signaling also activates anti-inflammatory mediators (110). This two-pronged activation of pro- and anti-inflammatory mediators serves to rapidly clear infection while limiting the overall level of immune system activation; however, this coordinated activation of pro- and anti-inflammatory mediators by TLR4 in humans is somewhat different in the horse (35).

Based on differences in equine TLR4-LPS signaling, the equine monocyte response to LPS is more pro-inflammatory than that of other species. The primary cause of this difference is that LPS-activation of TLR4 primarily signals through MyD88, with minimal activation of TRIF-mediated gene expression, as demonstrated in experiments with equine monocytes (50). As a result of MyD88 signaling, LPS stimulation of equine monocytes elicits a rapid and short-lived induction of TNFα mRNA, a rapid and more sustained expression of IL-1β mRNA, and slower but sustained induction of IL-6 mRNA. After an approximately 4-h delay, IL-10 mRNA expression is also induced, although the level of induction is much lower than that seen with TLR3 agonists such as double stranded viral DNA. The result is stronger signaling from the MyD88 pathway, which is regarded as pro-inflammatory, and diminished signaling from the TRIF pathway, which is considered anti-inflammatory. In other species, PI(3)Kδ regulates the switch in TLR4 signaling between MyD88 and TRIF, but this event does not seem to occur in equine monocytes. These attributes of TLR4 signaling specific for equine monocytes, taken together with their remarkable sensitivity to “nM” concentrations of LPS, provide a scientific basis for the marked inflammatory response that horses experience in response to endotoxin exposure (50).

### Cytokines in Sepsis

The focus of early human sepsis research was the SIRS, which was believed to be an overwhelming and unchecked spike in the host's pro-inflammatory immune response (96). Although SIRS can occur in response to trauma, anaphylaxis, ischemia, hemorrhage, and sterile inflammation (i.e., pancreatitis), in the case of sepsis the inciting event is documented or suspected infection. High levels of circulating pro-inflammatory cytokines, such as TNFα, IL-1, and interleukin-6 (IL-6), were cited as evidence of SIRS in both septic human patients and laboratory animals with induced sepsis. The belief that sepsis was a state of hyper-inflammation led to numerous clinical trials investigating agents that would block, neutralize, remove, or attenuate various pro-inflammatory mediators. As the majority of these studies failed, the proposed hyper-inflammatory pathogenesis of sepsis was re-examined. At that time, based on new evidence of immunosuppression/immunoparalysis in septic patients (111), it was proposed that SIRS was only the initial phase of sepsis followed by a compensatory anti-inflammatory response syndrome (CARS), leading to adverse events such as increased rate of nosocomial infections (96). Hallmark characteristics of CARS were cited as diminished expression of major histocompatibility complex class II molecules on circulating monocytes (mHLA- DR), massive apoptosis of circulating lymphocytes and elevated plasma levels of the anti-inflammatory cytokine IL-10 (112). This notion of a multi-modal or two-phase inflammatory response to sepsis has since been rejected and replaced by evidence of an integrated, highly mixed anti-inflammatory response syndrome (MARS) (113).

The cytokine features of equine sepsis and critical illness are currently the subject of intense study. At this time, much of the inflammatory cytokine data available is based on models of equine sepsis, such as intravenous LPS infusion or oral administration of black walnut extract (BWE) or carbohydrate. Evidence from these models shows that, as in other species, horses initially produce TNFα in response to LPS (114–116). High levels of circulating TNFα are also present in horses with colic, a disease that often elicits a systemic inflammatory response. Further, marked increases in TNFα have been linked to a higher rate of mortality in cases of equine colic and sepsis in foals (117, 118). TNFα is known to induce the expression of interleukin (IL)-1β, and administration of these two cytokines together is enough to reproduce septic shock in animals (119, 120). In horses, IL-1β elevations have been documented in foals with naturally occurring sepsis and in adult horses following LPS infusion (79, 121).

Another important cytokine during sepsis in humans and horses is IL-6, which is produced after cells are exposed to TNFα and IL-1β. While IL-6 is not considered to be a pro-inflammatory cytokine, neither is it considered anti-inflammatory. LPS infusion in adult horses causes a significant increase in circulating levels of IL-6 (121). In one report on neonatal foals with naturally occurring sepsis, IL-6 levels were lower than healthy age-matched controls (80). These investigators provide evidence that healthy foals acquire a significant amount of IL-6 from the passive transfer of immunity. In a different report, IL-6 gene expression was significantly increased in non-surviving septic foals compared to foals that survived sepsis (122). Because these two studies examined either IL-6 protein or mRNA, but not both, their apparently conflicting results are difficult to resolve. In human sepsis trials, persistently elevated levels of IL-6 correlated with disease severity and an increased risk of death (111). However, in foals evidence suggests that IL-6 protein is lower in the blood of septic vs. healthy foals and that the IL6:IL10 ratio, which was higher in the healthy age matched foals compared to septic foals, may be a useful prognostic indicator for neonatal septicemia (80).

The most commonly investigated anti-inflammatory cytokine in people and horses is IL-10, although other anti-inflammatory mediators include IL-4, IL-11, IL-13, transforming growth factor β, soluble TNF receptors and IL-1 receptor antagonist (87, 123). Anti-inflammatory cytokines offer a counterbalance to the pro-inflammatory signals, resulting in suppressed production of IL-1β, TNFα and chemokines and decreasing vascular adhesion molecules. Increased levels of IL-10 in human patients with sepsis is associated with poorer outcomes and an increased risk of MODS and death (124–126). In equine neonatal sepsis, significantly elevated levels of IL-10 have been reported in non-survivors compared to survivors (122). However, another study compared levels of circulating IL-10 between septic foals and healthy controls and found no difference (80). Whether or not IL-10 levels correlate with outcome in adult equine patients with naturally occurring sepsis has yet to be investigated.

## Neutrophils in Health and Sepsis

In 1882, Elie Metchnikoff observed recruitment of phagocytic cells to sites of injury in starfish embryos and hypothesized that they (i.e., neutrophils) were involved in microbe digestion (127). This observation began more than a century of research that continues to examine the role of neutrophils in host defense, host injury, microbial killing, resolution of inflammation, immunodeficiency and tissue healing. The goal of this section of the review is to cover broad topics of neutrophil physiology that are relevant to the pathophysiology of sepsis; the reader is referred elsewhere for details regarding the molecular mechanisms of sepsis and a more detailed examination of neutrophil signaling (10, 128).

### The First Responder

Neutrophils are essential for host defense against bacteria. Patients that are neutropenic or have leukocyte adhesion deficiency are at increased risk for bacterial infection (129). On the other hand, neutrophil effector functions, such as release of reactive oxygen species (ROS) and extracellular traps (NETs), can be very damaging to host tissue in a number of diseases including sepsis. Therefore, neutrophils are potential targets for combating sepsis-associated organ damage. A better understanding of neutrophil functions and molecular regulators of neutrophil responses could lead to novel targets for therapeutic modulation of neutrophil dysfunction in sepsis.

There are several key features of neutrophils that make them particularly effective at responding to bacterial invasion. (1) Neutrophils are the most abundant leukocyte in systemic circulation, with 2.9–8.5 × 10^9^ cells/l circulating in healthy adult horses, and 2.5–7.5 × 10^9^ cells/l in adult humans. Importantly, these circulating neutrophils are maintained in a quiescent state until they receive signals for recruitment and activation. This is an important safety mechanism that normally keeps the destructive power of neutrophils in check. (2) Large numbers of neutrophils are rapidly recruited to circulation from the reserves of neutrophils located in the bone marrow. This is particularly important, considering the lifespan of terminally differentiated neutrophils is assumed to be relatively short (i.e., half-life of 10.5 h in the horse and 8 h in humans) (130–132), although recent evidence that the average neutrophil lifespan is 5.4 days has cast doubt on that assumption (133). (3) Under physiologic conditions, neutrophils are found both in circulation as well as in marginated pools in the spleen, liver and lung (134, 135). Although the reason for the concentration of neutrophils in these organs remains uncertain, it is postulated that organ-neutrophils are acting as sentinels, providing constant surveillance for the detection of, and defense against, microbial invasion, and tissue damage. (4) In response to chemotactic stimuli, neutrophils can migrate at extremely fast velocities (up to 12 um/min), meaning they have a tissue target response time as short as 3 h after initial insult. (5) Neutrophils are equipped with a remarkable contingent of surface receptors and adhesion molecules that facilitate activation and endothelial transmigration and culminate in the arrival of neutrophils at remote sites of tissue injury or infection. All these properties make neutrophils uniquely suited to provide a rapid response to bacterial invasion in the host.

### Neutrophil Recruitment

Damage to tissues, whether the result of sterile injury or pathogenic invasion, is recognized by immune cells such as macrophages and mast cells as well as by stromal cells (136–138). Characteristic stimuli, including PAMPs and DAMPs, activate these cells to release pro-inflammatory mediators such as IL-1β and TNFα as well as chemoattractants for neutrophils (139, 140). The local release of these mediators causes the neighboring vascular endothelium to increase endothelial expression of P-selectin and then later E-selectin and ICAM-1 (141–143). Neutrophils passing the area of activated endothelium enter a reversible adhesion cascade that consists of six steps, mediated by specific ligand-receptor interactions and molecular mechanisms. The initial steps of the leukocyte adhesion cascade are capture and rolling. They are mediated by induced endothelial expression of P- and E-selectin and neutrophil-expressed L-selectins and E-selectin ligand (ESL-1), P-selectin ligand (PSGL-1) and CD44, which are expressed constitutively (144, 145). The strength of these selectin-mediated attachments only develops during the shear stress that neutrophils experience during laminar blood flow (146, 147). Also important for the progression of neutrophil adhesion to the blood vessel wall is the presentation of endothelial-bound chemokines to their respective G-protein coupled receptors (GPCRs) on the neutrophil cell surface. GPCR signaling leads to “inside-out” activation of neutrophil β2-integrins (148). This activation of neutrophil β2-integrins Mac-1 (CD11b, αMβ2) and LFA-1 (CD11a, αLβ2) facilitates adhesion to their respective ligands on the endothelial surface—ICAM-1 and ICAM-2—which leads to β2-intgerin “outside-in” activation and subsequent slow rolling, arrest, and finally adhesion strengthening. Following adhesion strengthening, neutrophils physically prepare for their transendothelial journey, taking on a characteristic polarized appearance with a leading edge lamellipodium and a trailing edge uropod (149). Using β2-integrin-dependent mechanisms, reviewed in detail elsewhere (150), neutrophils traverse the endothelium via transcellular or paracellular routes, cross the basement membrane and home in on sites of bacterial invasion.

### Chemotaxis

Circulating neutrophils, initially distant from the primary site of pathogen invasion, are recruited to the area of tissue infection by an uncontested gradient of endogenously produced host chemokines (i.e., interleukin-8 and leukotriene-B4) known as intermediate chemoattractants. As neutrophils cross the endothelium to enter the actual site of infection, they must “ignore” the chemokine gradients of diffusely inflamed host tissue in order to “target” invading microorganisms. End-stage chemoattractants derived from bacterial peptides and host complement factors, such as N-Formyl-methionyl-leucyl-phenylalanine (fMLP) and C5a, signal neutrophils to converge at the point of bacterial invasion in order to phagocytose foreign material and release their arsenal of antimicrobial agents including oxidants, proteinases, and cationic peptides (147).

Chemoattractant receptors play a vital role in translating the spatial cues of the chemoattractant gradient to the cellular machinery that drives neutrophil locomotion. Neutrophils “sense” these chemoattractant gradients by virtue of independent GPCRs and “prioritize” intermediate and end-stage chemoattractant signals using the phosphatidylinositol-3-OH kinase (PI(3)K) and tensin homolog (PTEN) pathway and the p38 MAPK pathway, respectively (151, 152). With damage to the machinery of migration (as occurs in sepsis), neutrophils lose their “compass,” fail to control infection and become a liability to their host.

### Abnormal Neutrophil Numbers in Sepsis

Altered numbers of circulating white blood cells are a defining characteristic of SIRS and sepsis (32). As the most abundant white blood cell in circulation, neutrophils play a significant role in determining whether patient WBC counts fall outside a normal reference range. During LPS infusion models of SIRS, circulating neutrophil numbers in adult horses fall significantly within the first 2–4 h (neutropenia) and then rebound to increased numbers (neutrophilia) (153, 154). Blood neutrophils are also significantly increased 18–20 h after oligofructose-induced colitis in adult horses (155). Changes in numbers of circulating neutrophils during SIRS and sepsis are attributed to mobilization signals from cytokines, bacterial products and other inflammatory mediators (156). Additionally, under these conditions the life span of neutrophils is altered. In healthy states, neutrophils are removed from circulation following intrinsic activation of apoptosis and macrophage phagocytosis in the spleen, liver and bone marrow (157); but in human neutrophils exposed to bacterial LPS (158) or neutrophils from patients with sepsis (159), apoptosis is delayed. This is also true in horses. Horses with strangulating intestinal lesions have a lower proportion of circulating apoptotic neutrophils than horses that underwent surgical arthroscopy (160). Recent investigations also show that induced oligofructose colitis in horses, which causes severe diarrhea and SIRS, results in a significant delay in neutrophil apoptosis *ex vivo*. These investigators went on to show that LPS treatment delays apoptosis of equine neutrophils *in vitro* in a dose-dependent manner (161), and that delayed apoptosis is attributed to caspase-9 activity but not caspase-3 or caspase-8 (161). These authors propose that the prolonged life span of neutrophils in endotoxemic horses may contribute to the development of SIRS.

Numbers of immature, or band, neutrophils are also altered during sepsis, and increase in these cells is known as a “left shift.” A “left shift” is significant because compared to mature neutrophils, immature neutrophils have a relatively longer life span and are resistant to spontaneous apoptosis, have higher basal levels of pro-inflammatory mediators compared to anti-inflammatory mediators (i.e., TNFα/IL-10 ratio), demonstrate reduced migration in response to chemoattractants, produce less ROS, and have decreased phagocytic and bactericidal activity (162, 163). An increase in the percentage of immature granulocytes (IG) is a marker of severe infection (164), has been associated with sepsis diagnosis (165) and is associated with mortality in neonatal sepsis (166). A recent prospective, observational study shows that in human ICU patients, total IG count discriminates between infected and non-infected patients with a sensitivity of 89.2% and a specificity of 76.4% in the first 48 h of SIRS. Additionally, IG count was more indicative of infection than other biomarkers including C-reactive protein, lipopolysaccharide binding protein (LBP) and IL-6. The IG count was not, however, a good prognostic marker for mortality in this patient population (167). Similar results were reported for the delta neutrophil index (DNI), an automated estimate of IG count, in septic dogs (168). DNI was significantly higher in dogs with sepsis compared to dogs with immune-mediated hemolytic anemia (IMHA) and healthy dogs and significantly higher in dogs with septic shock compared to septic dogs without circulatory failure; however, no differences were detected between survivors and non-survivors (168). In a recent prospective observational study of adult equine emergency patients, the presence of band neutrophils on blood smear at admission was associated with SIRS and with poor outcome (28). Mean number of band neutrophils was also significantly increased in horses with strangulating intestinal lesions compared with control horses (169) and in neonatal foals diagnosed with sepsis (170). The association of automated IG count with SIRS severity, sepsis and outcome has not been investigated in horses.

### Altered Neutrophil Rigidity and Capillary Bed Sequestration

During healthy conditions, normal neutrophils with a diameter of ~ 7–9 μm deform in order to pass through capillaries with an average diameter of 5–6 μm. In human patients with sepsis, neutrophil deformability is decreased, and neutrophils trapped in the capillary beds alter blood flow and release oxygen radicals and proteases that contribute to organ damage (171). *In vitro* studies have shown that decreased neutrophil deformability in response to fMLP or TNFα is associated with the appearance of a submembrane ring of F-actin (172). It has been reported that neutrophil rigidity increases proportionally with sepsis severity (173) and that this leads to an accumulation of neutrophils in the capillary beds of lung and liver sinusoids. In a rat model of bacterial pneumonia, neutrophils with submembrane F-actin were preferentially retained over neutrophils without F-actin during passage through the lungs (174). Decreased neutrophil deformability has also been detected in LPS-stimulated equine neutrophils (175) as well as in equine patients with conditions that cause SIRS, including colic due to inflammatory bowel disease and intestinal strangulation (169). In horses with intestinal strangulation, changes in neutrophil deformability, along with size, and granularity, were correlated with poor outcome (169). It is unknown whether alterations in actin structure contribute to neutrophil accumulation within the laminae, or other organs, of horses with sepsis.

### Altered Neutrophil Migration

The neutrophil's response to inflammation is mediated in large part by chemokines and cytokines, which signal neutrophils through GPCRs. During sepsis, the expression of these neutrophil agonists can be aberrantly increased. This can reduce neutrophil responsiveness in at least two ways. With a high rate of ligand binding, GPCRs are functionally desensitized due to a lack of gradient sensing ability (176). Additionally, GPCR-ligand binding leads to receptor internalization, which also effectively blunts GPCR-mediated neutrophil activation. These mechanisms are recognized clinically in septic patients, whose neutrophils have decreased surface expression of CXCR2 (also known as the IL-8 receptor) (177). In mice, sepsis has also been shown to upregulate the CCR2 chemokine receptor (178). The result of this upregulation is an increase in random neutrophil migration, an accumulation of neutrophils in remote organs and a failure of neutrophils to accumulate at the focus of infection. In this study, genetic, or pharmacologic inhibition of CCR2 protected mice from mortality in a cecal ligation puncture (CLP) sepsis model. In contrast to the lack of CXCR2 function that impacts neutrophil function during sepsis, the effects of CCR2 were associated with increased function, demonstrated by the finding that severity of patient illness correlated positively with increasing neutrophil chemotaxis to CCR2 ligands. The authors conclude that CCR2 may help drive the inappropriate infiltration of neutrophils into remote organs during sepsis. Interestingly, the altered expression patterns and functions of CXCR2 and CCR2 seen during sepsis are driven by TLR activation in neutrophils (178, 179). It is likely that both of these alterations in GPCR-mediated neutrophil function are contributing to dysfunctional migration of neutrophils during sepsis.

Evidence suggests that increased circulating levels of cytokines and chemokines may hinder normal neutrophil recruitment from the vasculature. Patients with sepsis frequently have elevated levels of IL-8, C5a, and TNFα in their blood (180). Elevated levels of TNFα have also been documented in foals with presumed “septicemia” (118). *In vitro*, IL-8 pretreatment led to reduced neutrophil migration across endothelial monolayers (181), and *in vitro* exposure of neutrophils to C5a concentrations comparable to those in the blood of septic patients completely paralyzed the neutrophil migrational response (112, 182). Elevated TNFα in the blood of septic patients decreases neutrophil migration, inhibits neutrophil apoptosis, enhances neutrophil priming, and production of reactive oxygen species and suppresses neutrophil CXCR2 expression (158, 183, 184). In *in vitro* studies, Brooks et al. showed that the chemoattractant IL-8 is able to attenuate migration of equine neutrophils to platelet-activating factor (PAF) and, to a lesser extent, LTB4, in a CXCL2-dependent manner. These authors conclude that this effect could lead to trapping of activated neutrophils at sites of inflammation *in vivo* (185). The increased presence of primed neutrophils within the vasculature, which has been demonstrated in septic patients, may extend neutrophil-endothelial interactions and ultimately contribute to vascular and organ damage.

### Altered Effector Functions

In order to combat pathogens, neutrophils produce inflammatory cytokines and chemokines, generate reactive oxygen species, phagocytose pathogens and release NETs (186–188). In neutrophils from septic patients, all these functions can be altered, but the extent of these alterations, as well as whether the functions are enhanced or suppressed, varies depending on the sepsis model being studied, the timing and the individual patient.

In one recent study, Tang et al. used microarray analysis to profile the gene-expression of neutrophils collected from septic patients within 24 h of hospital admission (189). These results document the downregulation of inflammatory response genes, immune modulation genes, and genes required for oxidant production. This is consistent with evidence of progressive impairment of oxidant production in murine neutrophils during Pseudomonas sepsis (190) and the impaired neutrophil phagocytic activity that has been correlated with poor outcomes in patients with sepsis (191). However, the dampening of neutrophil effector functions at the gene expression level is not indicative of complete immunosuppression because peripheral blood leukocytes from septic patients have substantial induction of several antimicrobial genes (186). It has been suggested that this divergence in the regulation of host defense genes is supportive evidence for reprogramming of neutrophil effector functions during sepsis.

The evidence for altered neutrophil phagocytosis during sepsis is variable. On one hand, human patients with severe sepsis and poor outcomes had decreased neutrophil phagocytic activity within the initial 24 h of hospital admission (191). On the other hand, neutrophils from septic patients show enhanced internalization and destruction of microorganisms, and neutrophils from septic foals do not differ significantly from healthy foals in phagocytic ability (192). Unfortunately, intact phagocytic function is not helpful if neutrophils fail to arrive at the focus of infection, and several different animal studies have shown that a lack of neutrophil migration into infectious sites is associated with reduced survival (176). In addition to the blunting of neutrophil chemotaxis seen with excess levels of circulating chemotactic mediators, researchers also attribute this failure of migration to decreased rolling and adhesion of neutrophils to endothelium, observed during CLP sepsis in mice (193). On the surface, it would seem difficult to resolve this observation with the evidence that neutrophil accumulation in tissue is contributing to sepsis-associated organ injury (i.e., MODS, laminitis). Clearly, when it comes to altered neutrophil migration during sepsis, the multiple mechanisms that differentially affect the neutrophil's ability to traverse the endothelium are likely impacted by the proximity of that endothelium to sites of infection and specific organs.

In order to destroy invading microorganisms, activated neutrophils produce ROS such as superoxide (O_2_-) through a process known as respiratory burst. In order to generate and release ROS, neutrophils must assemble a five-subunit enzyme complex known as NADPH at either the phagosome or plasma membrane. Within the resting neutrophil, three of the subunits (p40PHOX, p47PHOX, and p67PHOX) are consistently present within the cytosol, while the other two components (p22PHOX and gp91PHOX) are located within the membranes of the secretory vesicles and specific granules. It is only when neutrophils are fully activated that the components assemble and ROS are released. This is an important protective mechanism for the host, as the release of ROS can damage not only microorganisms but also host tissue. This is true in septic patients, whose neutrophils have an enhanced ability to generate reactive oxygen species compared to healthy people, and whose plasma has been shown to cause ROS generation by human umbilical vein endothelial cells, which has been correlated with organ dysfunction and mortality. Increased neutrophil ROS generation detected in septic patients on day 0 is decreased in survivors by day 7, whereas it remains elevated in non-survivors (194).

Neutrophil extracellular traps are the most recently discovered tool in the neutrophil's arsenal of host defense mechanisms (195). NETs are formed when elastase, released from azurophlic granules, causes decondensation of nuclear chromatin, which is then extruded (along with histones, myeloperoxidase, neutrophil elastase, cathepsin G, and antimicrobial proteins) extracellularly. NETs are released from neutrophils in response to cytokines (i.e., IL-8), microbial components (i.e., LPS), phorbol esters (i.e., PMA), and activated platelets and endothelial cells. NETs enhance neutrophil bacterial killing by physically capturing bacteria and bringing them into close proximity with antimicrobial proteins. Indeed, the effectiveness of NETs in killing a wide variety of Gram-positive and Gram-negative bacteria, as well as fungi (*Candida albicans*) and protozoa (*Leishmania amazonensis*), has been demonstrated both *in vivo* and under flow conditions *in vitro* (196). While NETs are clearly beneficial for host defense, widespread release of NETs may have a role in organ injury during sepsis (197). In various studies, NETs have been shown to contribute to hepatocellular injury, damage endothelial cells and instigate fibrin deposition and thrombus formation (198, 199). An indirect measure of neutrophil NETs in systemic circulation is plasma cell-free DNA (cfDNA). In a CLP mouse model, and in naturally occurring pediatric and canine sepsis, the ratio of cfDNA to neutrophils is increased with sepsis (200–202). A recent human prospective observational cohort study determined that *ex vivo* neutrophil NETosis was impaired in patients with severe sepsis compared to septic patients and controls and that reduced NETosis *ex vivo* was associated with poorer outcomes in septic patients (203). To date, investigations of NETosis of equine neutrophils has been limited to reproductive and respiratory research (204–208). The importance of NETs in equine sepsis pathophysiology, diagnosis, and treatment remains to be determined.

### Neutrophil Mediated Organ Damage

After the successful elimination of bacteria, neutrophils undergo apoptosis, or programmed cell death (209), but during sepsis, neutrophil apoptosis is delayed (see previous). This may contribute to the accumulation of neutrophils in the organs of patients with sepsis, an occurrence that is commonly documented in animal models of sepsis as well as during autopsies of human patients that died as a result of sepsis-induced multiple organ failure (210, 211). In humans with sepsis-mediated organ dysfunction, the lungs are the most common organ to undergo remote or secondary injury (212). This complication is referred to as acute lung injury (ALI) or acute respiratory distress syndrome (ARDS). Among the key players in the pathophysiology of ALI/ARDS, neutrophils are considered to have a crucial role in disease progression and outcome (213). During the initial exudative phase of ALI and ARDS, several pro-inflammatory mediators (i.e., IL-8) initiate migration and pulmonary infiltration of neutrophils into the interstitial tissue, where they cause injury and breakdown of the pulmonary parenchyma. In patients with ARDS, the concentration of IL-8 and the concentration of neutrophils in the bronchoalveolar lavage (BAL) fluid correlate with ARDS severity and outcome (214), and severity of lung injury can be reduced by neutrophil depletion in mice (215).

In both neonatal and adult horses, naturally occurring, and experimentally induced systemic inflammation and/or sepsis has been associated with increased neutrophil accumulation in target organs and/or multiple organ dysfunction. Reports of organs affected include the lungs, heart, kidneys, and laminae (87, 216–220). However, in studies using experimental models of sepsis-related laminitis, the increased inflammatory cytokines and leukocytes detected in the liver, lung, and kidney did not affect function of these organs (221, 235). Because of the significance of equine laminitis, a great deal of interest and research has focused on determining the pathophysiology of sepsis-associated lamellar injury. Laminitis experts have compared sepsis-mediated laminar injury in horses to sepsis-induced organ failure in people (222). This comparison is based on the observation that laminar changes during the prodromal and acute stages of induced laminitis in horses are similar to the changes observed in organs at risk of failure during human sepsis. These changes include increases in tissue pro-inflammatory cytokines, leukocyte activation, endothelial activation, and neutrophil tissue infiltration and accumulation (223, 224). Neutrophils are not only observed as being physically present in the laminae, they also contribute to the release of myeloperoxidase BWE model and epithelial injury (BWE- and carbohydrate overload-model) (218, 225, 236). Whether or not neutrophils are a target for decreasing the risk of sepsis-associated laminitis in the horse is a question that remains to be answered. Digital cryotherapy, achieved by applying ice to the distal limbs by bucket or boot, inhibits the accumulation of neutrophils in the laminae as well as the upregulation of pro-inflammatory cytokines (226–228). Icing the equine digit also decreases lamellar gene expression of the neutrophil chemokines CXCL1, −6 and −8 (229) and decreases expression of the leukocyte adhesion molecules ICAM and E-selectin (228). In numerous animal studies, neutrophil depletion, or targeted inhibition of neutrophil-endothelial adhesion has prevented the development of sepsis-associated organ damage (230). This approach seems to be a reasonable next step in beginning to unravel the role of neutrophils in the pathophysiology of equine laminitis.

## Early Goal Directed Therapy in Sepsis

Of all of the potential therapies investigated for the treatment of human sepsis over the past 20 years, the most significant positive impact on human mortality came from research in the early 1990s which established early goal-directed therapy (EGDT) (2). EGDT is a systematic approach to resuscitation of the severely septic and septic shock patient, and it is comprised of early identification of high-risk patients, culture collection, appropriate antimicrobial therapy, and infection source control, followed by aggressive management of hemodynamic parameters. Its principles aim to optimize oxygen delivery to tissues through the rapid stabilization of cardiac preload, afterload and contractility (231). Therapies utilized to achieve this goal include fluids, vasopressors, or vasodilators (as indicated) and the option of intubation and mechanical ventilation. Routinely measured patient parameters used to guide therapy include central venous pressure (CVP), mean arterial pressure (MAP), mixed venous oxygen saturation (SvO2), and urine output.

After a 3-year randomized controlled clinical trial (1997–2000) and more than a decade of national and international reliability and feasibility testing, EGDT became an established component of the “sepsis resuscitation bundle ” for the Surviving Sepsis Campaign (SSC) (232). In an attempt to improve upon EGDT, in 2014 and 2015 three different groups published trials with versions of EGDT called ProCESS (Protocol-Based Care for Early Septic Shock), ARISE (Australian Resuscitation in Sepsis Evaluation) and ProMISe (Protocolized Management in Sepsis) (231). These trials were referred to as the “trio of EGDT trials.” Compared to the 30.5% mortality rate of the 1997–2000 EGDT trial, the reported sepsis mortality rates of 18.2, 14.5, and 25.6% for the ProCESS, ARISE, and ProMISe trials, respectively, appeared to be a significant improvement. However, due to differences in study design between the “trio trials” and the EGDT trial, such as time to patient enrollment, high rates of eligible patient exclusion, significant differences in hemodynamic phenotype of patients at baseline and an overall declining sepsis mortality rate in the United States, it is very difficult to compare the results of the “trio trials” to the original EGDT trial. Differences between the 1997–2000 EGDT trial and the “trio of EGDT trials” included: screening using SIRS, fluid challenge and lactate for inclusion in the “trio trials” compared to no screening criteria in the EGDT trial, lower initial fluid volume of 1 liter bolus in the “trio trials” compared to the 20–30 mL/kg in the EGDT trial, majority of care provided in ICU for “trio trials” compared to emergency department care for the EGDT trial, lower rate of mechanical ventilation for the “trio trials,” 50% more vasopressor use in the “trio trials” compared to the EGDT trial, steroid use of 8–37% in the “trio trials” compared to no steroids in the EGDT trial and a mortality benefit if antibiotics were administrated within the first 6 h in the “trio trials” (231).

The use of EGDT in equine medicine and critical care is gaining attention (233). While SvO2 is not routinely measured in equine patients at this time, other measures of tissue oxygenation include pH, base excess (BE) and lactate. In human medicine, “resuscitation endpoints” for EGDT are clearly defined. Although similar endpoints are not defined for the horse, it is likely that agreement on such parameters will aid equine critical care specialists with the application of these therapeutic principles.

## Neutrophils as Therapeutic Targets for Sepsis

In the February 2014 issue of Nature Nanotechnology, Wang et al. demonstrate the prevention of vascular inflammation and ALI (in animal models of inflammation and sepsis, respectively) by nanoparticle targeting of neutrophils (234). The albumin-derived nanoparticles, loaded with the spleen tyrosine kinase (Syk) inhibitor piceatannol, were internalized preferentially by endothelial-adherent neutrophils via cell surface Fc receptors. Because Syk is required for β2-integrin “outside-in” signaling, these adherent neutrophils subsequently detached from the endothelium. Nanoparticles were not taken up by circulating neutrophils. This method of nanoparticle drug delivery is an important step in demonstrating the feasibility of inhibiting activated, pro-inflammatory neutrophils without inhibiting all neutrophils. This therapeutic strategy, and others like it, could lead to exciting impacts for the treatment of neutrophil-mediated organ injury and disease such as ALI, laminitis and sepsis.

## Conclusion

The pathophysiology of sepsis and its associated syndromes is extremely complex, and important components not covered in this review include the roles of endothelial damage, the acute phase response, coagulation, and regulatory T cell responses. Given the complexity of this disease and its significant impact on equine health, more research is needed to better understand basics in pathophysiology, to justify or reject current treatment protocols and to discover new therapeutic modalities. In order to accomplish these goals, equine health professionals need to establish reliable, consistent definitions of sepsis (and its associated syndromes), as well as means for gathering and sharing real incidence and survival data.

## Author Contributions

The author confirms being the sole contributor of this work and has approved it for publication.

### Conflict of Interest Statement

The author declares that the research was conducted in the absence of any commercial or financial relationships that could be construed as a potential conflict of interest.
